# Charactering the *ZFAND3* gene mapped in the sex-determining locus in hybrid tilapia (*Oreochromis spp*.)

**DOI:** 10.1038/srep25471

**Published:** 2016-05-03

**Authors:** Keyi Ma, Minghui Liao, Feng Liu, Baoqing Ye, Fei Sun, Gen Hua Yue

**Affiliations:** 1 Molecular Population Genetics and Breeding Group, Temasek Life Sciences Laboratory, 1 Research Link, National University of Singapore, Singapore 117604, Republic of Singapore; 2 Department of Biological Sciences, National University of Singapore, 14 Science Drive 4, Singapore 117543, Republic of Singapore; 3 School of Biological Sciences, Nanyang Technological University, 60 Nanyang Drive, Singapore 637551, Republic of Singapore

## Abstract

Zinc finger AN1-type domain 3 (*ZFAND3*) is essential for spermatogenesis in mice. However, its function in teleosts remains unclear. In this study, we characterized the *ZFAND3* gene (termed as *OsZFAND3*) in an important food fish, tilapia. The *OsZFAND3* cDNA sequence is 1,050 bp in length, containing an ORF of 615 bp, which encodes a putative peptide of 204 amino acid residues. Quantitative real-time PCR revealed that the *OsZFAND3* transcripts were exclusively expressed in the testis and ovary. *In situ* hybridization showed that the high expression of *OsZFAND3* transcripts was predominantly localized in the spermatocyte and spermatid. These results suggest that *OsZFAND3* is involved in male germ cell maturation. Three single nucleotide polymorphisms (SNPs) were detected in the introns of *OsZFAND3*. The *OsZFAND3* gene was mapped in the sex-determining locus on linkage group 1 (LG1). The three SNPs in the *OsZFAND3* gene were strictly associated with sex phenotype, suggesting that the *OsZFAND3* gene is tightly linked to the sex-determining locus. Our study provides new insights into the functions of the *OsZFAND3* gene in tilapia and a foundation for further detailed analysis of the *OsZFAND3* gene in sex determination and differentiation.

Zinc fingers are extremely abundant in higher eukaryotes^1^. As a kind of protein motif with finger-like protrusions, zinc fingers were first identified in a study of transcription in the African clawed frog, which revealed that the binding strength of a small transcription factor (TFIIIA) was due to the presence of zinc finger structures^2^. Since the TFIIIA was reported, the DNA-binding properties of zinc fingers have been explored^1^. Also, it has been later reported that zinc fingers can mediate protein-protein interactions^3^. Until now, numerous genes with varying functions have been verified to encode zinc finger proteins, including sex-determining and differentiation genes. For example, transformer-1 (*tra-1*) in the nematode *Caenorhabditis elegans*, which activates sex differentiation genes downstream, encodes two zinc fingers^4^; steroidogenic factor-1 (*SF*-1) in humans, which can cause XY sex reversal, contains two zinc fingers^5^; and different isoforms of male-specific fruitless (*fru*) in *Drosophila melanogaster* have diverse regulatory roles conferred by zinc finger domains^6^.

Among the important zinc fingers, the A20/AN1-type zinc finger protein family is conserved in animals and plants^7^. However, only a few A20/AN1-type proteins have been found in fish and mammals^7^. A20, which contains seven Cys2/Cys2 zinc fingers at its C-terminal, is firstly identified as a cytokine-inducible gene with the function of primary response in humans^8^. As an ubiquitin-like fusion protein, the AN1 domain was originally found at the C-terminus of AN1 type and localized to the animal hemisphere of *Xenopus laevis* eggs or in early embryos^9^. However, the function of AN1 domain had been unclear until the characterization of zinc finger AN1-type 3 (*ZFAND3*) in mice in 1999^10^. *ZFAND3*, also known as the testis-expressed sequence 27 (*Tex27*), was first isolated from the mouse testis and its expression is essential for spermatogenesis^10^. In humans, recent study showed that *ZFAND3* is also involved in type II diabetes^11^. Although molecular cloning of *ZFAND3* cDNA has been performed in vertebrates, such as reptiles^12^ and amphibians^9^, the function of *ZFAND3* remains largely unclear in fish.

Tilapia is the common name for a group of cichlid fishes native to both fresh and salt water in Africa and the Middle East. As one of the most important food fish in the world^13^, tilapia could provide a good source of animal protein and high-quality meat with minimal inputs and efficient conversion from plant material to fish biomass^14^. In most tilapia species, there is a significant difference in growth performance between males and females. Males grow much faster than females and reach a larger size after reaching maturity^14^. The best way to obtain a male monosex population is through genetic control by developing YY “super males” which can sire more male progenies^15^. This technology is therefore, reliable and consistent, and more and more sex-associated markers were identified and utilized in various tilapiine species. For example, microsatellite markers on linkage group (LG) 3 and LG1 were linked or associated with sex phenotype in blue tilapia, respectively^16^. Sex-determining loci were mapped to LG1 and LG23 in Nile tilapia (*Oreochromis niloticus*)^17^,^18^, LG3 in hybrid population of *O. niloticus *× *O. aureus*^19^, and LG1 and LG22 in Mozambique tilapia and red tilapia^13^. By using RAD sequencing, a set of SNP markers highly associated with phenotypic sex were identified on LG1 in the *O. niloticus* families^20^. Recently, whole genome sequencing was performed in *O. niloticus*, in which a potential 8.8 Mb inversion was identified in a sex-determining locus on LG1^21^. So far, one candidate sex-determining gene, *amh* on LG23, has been identified in *O. niloticus*^22^. Although our previous study showed that one sex-determining locus was mapped onto LG1 in a tilapia family^13^, no potential sex-determining gene has been identified on LG1 in this family yet.

The purpose of this study was to characterize the *OsZFAND3* gene, which was mapped onto the sex-determining locus on LG1 in the family of hybrid tilapia (*Oreochromis spp*.) constructed by crossing a Mozambique tilapia male and a red tilapia female. We found that the gene was expressed exclusively in the testis and ovary. *In situ* hybridization showed that the high expression of *OsZFAND3* transcripts was predominantly localized in the spermatocyte and spermatid. Additionally, three single nucleotide polymorphisms (SNPs), detected in the introns of *OsZFAND3*, were strictly associated with sex phenotypes. Our results suggest that *OsZFAND3* is tightly linked to the sex-determining locus, and it may play an essential role in male germ cell maturation.

## Results

### Characterization of *OsZFAND3* sequence and linkage mapping of the *OsZFAND3* gene

**Figure 1:**
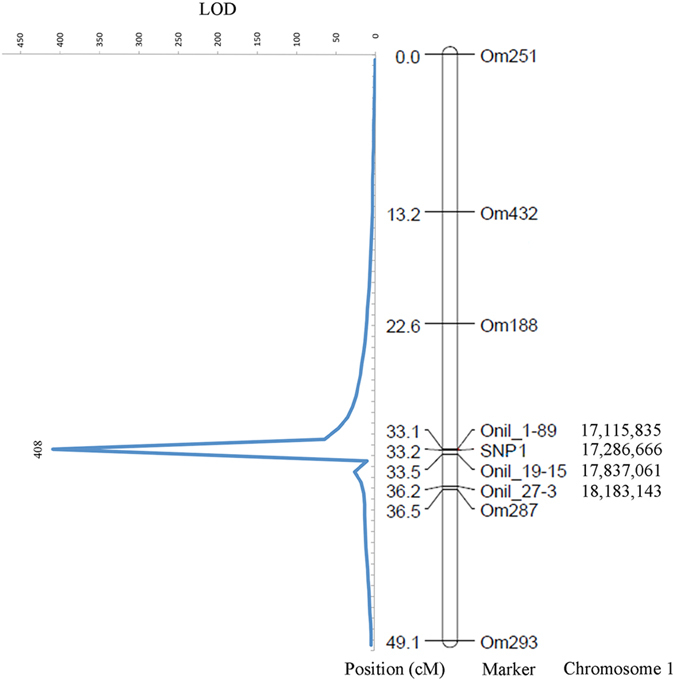
Locations of SNP1 and microsatellite markers on LG1 of hybrid tilapia and linkage mapping of *OsZFAND3*. Locations of the four markers (Onil_1-89, Onil_19-15, Onil_27-3 and SNP1) developed in this study are labelled in the genetic map in cM and anchored in the *O. niloticus* draft genome in bp.

Estimation of exon-intron boundaries by alignment of *OsZFAND3* cDNA with the genomic sequence identified six exons and five introns in total (Supplementary Fig. S1A). All the observed splice acceptor and donor sites were in accordance with the consensus GT-AG rules. The *OsZFAND3* cDNA sequence is 1,050 bp in length and contains an ORF of 615 bp, encoding a putative peptide of 204 amino acid residues (aa) (Supplementary Fig. S1B). The deduced amino acid sequence of *OsZFAND3* has an A20-like zinc finger domain located from Pro_19_ to Ala_41_ at the N-terminal and a Serine-rich region from Ser_102_ to Ser_164_. After searching against the cNLS Mapper, potential nuclear localization signal (NLS) with consensus peptide sequences “RSRKRCHRC” was found in the *OsZFAND3* protein. Targeted sequencing of the 279 tilapia F_1_ individuals of one family identified three SNPs (SNP1 T/C, SNP2 T/C and SNP3 A/G) in the *OsZFAND3* gene (Supplementary Fig. S1A). SNP1 and SNP2 were located in the first intron, while SNP3 was found in the fifth intron. All the females (n = 151) were homozygous T/T for SNP1, T/T for SNP2 and A/A for SNP3, and the males (n = 128) were heterozygous C/T for SNP1, C/T for SNP2 and G/A for SNP3, respectively (Supplementary Table S1). This indicates that there is no recombination between the SNP genotypes and the sex phenotypes among the 279 individuals. In the following study, SNP1 was selected for linkage mapping (Fig. 1). Using this SNP, which has a logarithm of odds (LOD) value of 408, *OsZFAND3* was mapped onto LG1, between microsatellite markers Onil_1-89 and Onil_19-15 (Fig. 1).

### Phylogenetic analysis of the *OsZFAND3* gene

The deduced amino acid sequence of *OsZFAND3* was compared with those of other vertebrates (Supplementary Table S2). *OsZFAND3* showed a high identity (69–99%) with the *ZFAND3* of fish (except *Danio rerio*) and relatively low identity (45–52%) with those of mammals, reptiles and birds. The data were further analyzed to construct the molecular phylogenetic tree using amino acid sequences of *ZFAND3* of selected species (Supplementary Fig. S2). The sequences could be divided into two clades, one of which included *ZFAND3* from the selected fish, and another one contained the ones from other vertebrates. As expected, the *OsZFAND3* and *O. niloticus ZFAND3* was clustered with each other, with a well-supported bootstrap value of 100%. In addition, the selected species were used to analyze the adaptive evolution of the *ZFAND3* gene. Pairwise estimates revealed that the dN/dS values among these species were less than 1 (Supplementary Table S3), suggesting that purifying selection could be playing an essential role in the evolution of the *ZFAND3* gene.

### Tissue distribution of the *OsZFAND3* transcripts and its expression patterns

**Figure 2:**
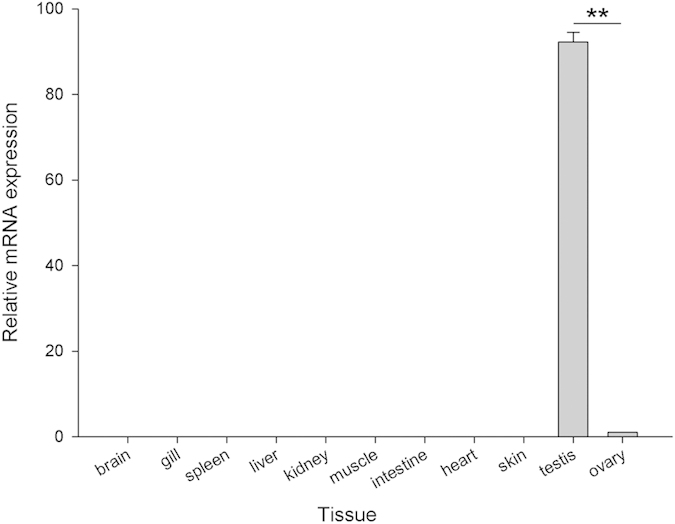
Tissue distribution of *OsZFAND3* mRNA in 11 tissues from three female and three male hybrid tilapia individuals using qPCR. Each bar indicates the mean + SE. ** Indicates that the difference is extremely significant.

The expression profiles of *OsZFAND3* transcripts in 11 tissues were analyzed with qPCR. As shown in Fig. 2, *OsZFAND3* transcriptional expression was observed exclusively in the testis and ovary. Moreover, the expression level of *OsZFAND3* transcripts in the testis was approximately 90 times than that in the ovary (*P* < 0.01). No product was detected from the other examined tissues or the negative control using deionized water as a template.

**Figure 3:**
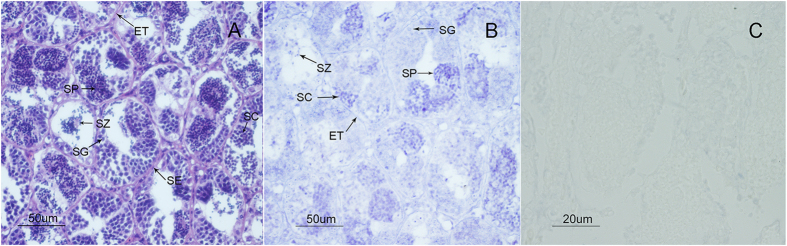
Expression pattern of the hybrid tilapia *OsZFAND3* transcripts as revealed by RNA *in situ* hybridization in the testis. (**A**) histological photograph of the testis development, and the section was stained with hematoxylin and eosin. (**B**) RNA *in situ* hybridization in the testis using the anti-sense probes, and (**C**) sense probes were used as the negative control. SE, sertoli cellls; ET, epithelia of the seminiferous tubule; SG, spermatogonium; SC, spermatocyte; SP, spermatid; SZ, spermatozoa.

Tilapia testis is composed of numerous seminiferous tubules which contain abundant germ cells at diverse developmental stages (Fig. 3). Each of the seminiferous tubules is surrounded by the epithelium where the sertoli cells are located (Fig. 3A). As the initial stage of spermatogenesis, the spermatogonium is the biggest in size of all the germ cells in the tilapia testis, located close to the inside edge of the seminiferous tubule (Fig. 3A). Through mitosis, the spermatogonium transforms into spermatocyte, which undergoes meiosis to produce spermatids, which in turn produce spermatozoa. In our study, the tilapia spermatozoa were found in the center of the seminiferous tubule (Fig. 3A). To examine the spatial expression pattern of *OsZFAND3* transcripts in the gonads, *in situ* hybridization was performed on the testis and ovary of the hybrid tilapia. However, we failed to detect the *OsZFAND3* transcripts in the ovary, possibly due to its low expression levels. At the beginning of spermatogenesis, the signal detected was very weak in most of the spermatogonia. At later stages of development, strong signals were detected in the spermatocytes and spermatids. Subsequently, weak signals could also appear in the spermatozoa (Fig. 3B). No signal was detected in the epithelia (Fig. 3B), and when using a sense RNA probe in the testis as a negative control (Fig. 3C).

### Subcellular localization of the OsZFAND3 protein

**Figure 4:**
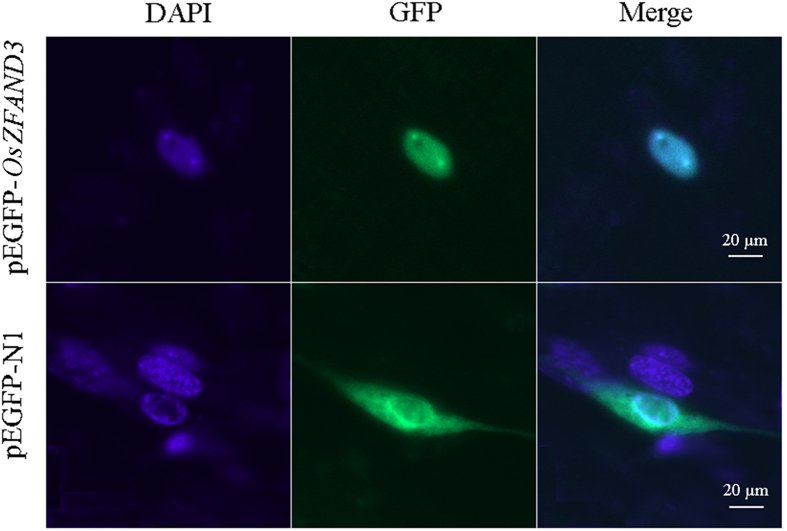
Intracellular localization of *OsZFAND3*. Tilapia nerve fibers cells were transiently transfected with pEGFP-N1 control vector (below row) and pEGFP-*OsZFAND3* (upper row). The localization of the nucleus was shown by DAPI staining.

To determine the subcellular localization of the OsZFAND3 *in vitro*, we detected the fluorescence using the inverted confocal after transient transfection of tilapia nerve fiber cells with recombinant plasmid pEGFP-*OsZFAND3*, and empty pEGFP-N1 vector was used as a control. As shown in Fig. 4, the green fluorescence was observed throughout the cytoplasm and nuclei in pEGFP-N1 transfected cells (lower row), while the green fluorescence of pEGFP-*OsZFAND3* overlapped exactly with the blue fluorescence of nuclei stained by DAPI, indicating that the OsZFAND3 protein was only localized in the nuclei in the pEGFP-*OsZFAND3* transfected cells (upper row).

## Discussion

In the present study, we reported the identification and mapping of the *OsZFAND3* gene in tilapiine species. Generally, *ZFAND3* protein tends to contain two zinc-finger domains, the A20 zinc-finger domain and the AN1 zinc-finger domain^12^. However, the deduced OsZFAND3 protein has an A20-like zinc finger domain and a Serine-rich region located at N-terminal and C-terminal, but has lost the AN1 zinc-finger domain (Supplementary Fig. S1B). This could affect the three-dimensional structure and function of the OsZFAND3 protein. As a stable domain in protein structure, the Serine-rich region is often considered as a protein-interaction motif^23^. Furthermore, two (T/S) PXX sequences, existing in the Serine-rich region of the deduced OsZFAND3 protein, could mediate DNA-protein interaction^12^. Animals exhibit high diversity of domain organization in the A20/AN1 zinc finger gene during evolution^24^, and the gain and loss of entire zinc finger domains could enable the appearance of functional divergence in proteins^25^. Compared to the *ZFAND3*s of the Japanese quail and the leopard gecko^12^, the missing AN1 zinc-finger domain in *OsZFAND3* leads to a large region of difference outside the zinc finger at N-terminal and may generate the function diversity of *ZFAND3* gene.

Vertebrates exhibit diverse sex determination systems^26^, resulting in different sex-determining genes depending on the species^26^,^27^,^28^,^29^,^30^. The tilapiine sex determination system is relatively complex^13^,^17^,^18^,^31^,^32^, as both XX/XY and ZW/ZZ sex-determination systems are known to occur in tilapia, implying that tilapia with divergent genetic backgrounds could have different sex chromosomes. In this study, the three identified SNPs in the *OsZFAND3* gene showed that our tilapia family might have an XX/XY sex determination system, which is consistent with the previous study in which the females were identified as homozygotes^13^,^17^. Due to prevalent interspecies crossing between the tilapiine species, the complexity of genetic backgrounds between families could be the main reason for this divergence of homozygosity or heterozygosity in both sexes. Additionally, various sex determination systems imply that different sex-determining genes may exist in tilapia families^16^.

One of the important characteristics of a sex-determining gene is its tight linkage with the non-recombinant part of the heterochromosome^33^. The three SNP loci of *OsZFAND3* are strictly associated with the sex phenotype on LG1. Hence it implied that *OsZFAND3* is tightly linked with the phenotype of sex in our tilapia family. However, to determine whether it is a potential sex-determining gene that plays an essential role in controlling testis differentiation, further work such as carrying out a transgene method should be performed. Additionally, the sex-determining region in the present study is very long (approximately 1.0 million bps), therefore, families of different genetic backgrounds and more offspring of tilapia are needed to fine map and detect whether recombination exists in this sex-determining locus in the future study.

According to the previous studies, *ZFAND3* transcripts were expressed in various tissues in plants, and represented most of the expression profile of A20/AN1-type zinc-finger family genes^7^. The same result was also found in the mouse, where the *ZFAND3* mRNA was expressed not only in the gonads, but also in the somatic tissues, such as kidney and liver^10^. In contrast to these studies, *OsZFAND3* exhibits quite a distinct expression pattern, being exclusively expressed in the testis and ovary according to qPCR analysis (Fig. 2), suggesting that it could be essential for maintaining the function of testis and ovary. The determination of expression levels of *OsZFAND3* transcripts in the testis was fully supported by the results of *in situ* hybridization. Although the hybridization signal was very weak in most of the spermatogonia, high expression of the transcripts was detected to be predominantly localized in the spermatocyte and spermatid germ cells. At the last stage of spermatogenesis, the weak signal then re-appeared in the spermatozoa (Fig. 3B). The best explanation of the differential distribution is that *OsZFAND3* could be related to the meiosis from the stage of spermatocyte to spermatozoa, suggesting that *OsZFAND3* could be involved in male germ cell maturation in tilapia.

Nuclear proteins typically contain NLS, directing nuclear import for the relative protein^34^. Unlike the classical NLS consensus isolated from nuclear import pathway^35^, other proteins with NLS consensus sequences have not been very clear so far. Although OsZFAND3 protein is exclusively localized in the nucleus, strongly suggesting that it is a nuclear protein, there was no NLS of *OsZFAND3* found after running the NLS search against the PredictProtein server. Since the database in the PredictProtein must undergo experimental verification, novel NLS could be missed, which may lead to false-negative result. In contrast to the PredictProtein, potential NLS was found in the OsZFAND3 protein after searching against the cNLS Mapper, indicating that OsZFAND3 is a nuclear protein with a new probable NLS.

As a confirmed family of transcription factors, SOX proteins usually contain NLSs^36^. Among the family, *SRY* and *Sox9* are critical regulators of the mammalian sex-determining pathway^30^,^36^. *OsZFAND3* is a sex-linked gene in tilapia with probable existing NLS, implying that it could be a functional transcription factor like the members in Sox family. However, future research such as yeast two-hybrid system and electrophoretic mobility shift assay should be performed to confirm this point.

In summary, we characterized the *OsZFAND3* cDNA sequence in the food fish tilapia. Although *OsZFAND3* transcriptional expression was observed exclusively in the testis and ovary, the expression level in the testis was approximately 90 times than that in the ovary. *In situ* hybridization showed that the high expression of *OsZFAND3* transcripts were predominantly localized in the spermatocyte and spermatid germ cells, and the weak signal appeared in the spermatozoa. Taken together, *OsZFAND3* could be involved in male germ cell maturation in tilapia. In terms of the SNP genotype, the *OsZFAND3* gene is tightly linked to the sex-determining locus on LG1 in our hybrid tilapia family constructed by crossing a Mozambique tilapia male and a red tilapia female. Our study sheds new insights on the function of *OsZFAND3* in tilapia and provides the basis information for further detailed analysis of its function in sex determination and differentiation.

## Materials and Methods

### Ethics statement

All handling of fish was carried out in accordance with the guidelines on the care and use of animals for scientific purposes set up by the Institutional Animal Care and Use Committee (IACUC) of the Temasek Life Sciences Laboratory, Singapore. The IACUC approved this study with approval number, TLL (F)-12-004.

### Fish rearing and tissue sampling

A total of 279 hybrid tilapia F_1_ offspring from crossing a Mozambique tilapia male and a red tilapia female, at the age of 140 days post-hatch (dph) with an average body weight of ~120.0 g, were used in this study. This tilapia family was used in our previous study, and one sex-determining locus was mapped on LG1^13^. Tilapia individuals were reared in a three-ton tank in the animal house of the marine fish facility of Temasek Life Sciences Laboratory, and fed twice daily with commercial pellet feed (Biomar, Nersac, France). Brain, gill, spleen, liver, kidney, muscle, intestine, heart, skin, testis and ovary tissues were collected from three males and three females, respectively. The samples were immediately frozen in liquid nitrogen and stored at −80 °C until total RNA extraction. For histological observation and *in situ* hybridization, the ovarian and testis samples were fixed in 4% paraformaldehyde solution (PFA) overnight at 4 °C.

### Sequence analyse of the *ZFAND3* gene

The *ZFAND3* gDNA (Genbank accession no. NC_022199.1) and cDNA (XP_005447615.1) sequences of Nile tilapia were obtained from the NCBI database. Primers were designed based on this gDNA sequence to confirm the *OsZFAND3* cDNA sequence, and identify SNPs of the *OsZFAND3* in the hybrid tilapia (Supplementary Table S4). All primers were designed by Primer3Plus (http://primer3plus.com/). The open reading frame (ORF) of *OsZFAND3* was identified using the ORF Finder (http://www.ncbi.nlm.nih.gov/projects/gorf/). The NCBI alignment search tool, BlastN, was used to identify homologous sequences in GenBank (http://blast.ncbi.nlm.nih.gov/Blast.cgi). Sequences were aligned using CLUSTALW 2.0^37^. Based on the deduced full-length *ZFAND3* amino acid sequence alignments, a phylogenetic tree was constructed by the Neighbor-Joining (NJ) algorithm method embedded in the MEGA 6.0 software^38^. The reliability of the analysis was assessed by 1000 bootstrap searches. Maximum likelihood analysis was employed to detect adaptive evolution in the *ZFAND3* gene using the YN00 program in the PAML X package^39^. The nuclear localization signal (NLS) of *OsZFAND3* was identified using the PredictProtein server (https://www.predictprotein.org/) and cNLS Mapper (http://nls-mapper.iab.keio.ac.jp/).

### Genotyping of microsatellite markers

In order to fine map the sex-determining locus on LG1, gDNA was extracted from the caudal fin of the 279 tilapia F_1_ individuals mentioned above, according to the protocol developed previously^40^, and primers of three microsatellite markers (Onil_1-89, Onil_19-15, Onil_27-3, see details in Supplementary Table S4) flanking the sex-determining locus were designed in this study. The PCR amplification for each individual sample was performed in a 25 μL volume, containing 10 ng gDNA, 1 × PCR buffer, 80 μM of each dNTPs, 0.2 μM forward and reverse primers and 1 U Taq DNA polymerase (Thermo Fisher Scientific, MA, USA). The reactions were carried out in a thermal cycler (MJ Research, CA, USA) using the following programme: one cycle of 3 min at 94 °C, 38 cycles of 30 sec at 94 °C, 30 sec at 55 °C and 30 sec at 72 °C, followed by a prolonged extension of 8 min at 72 °C. The PCR products were resolved on an ABI 3730XL DNA sequencer (Applied Biosystems, CA, USA) and genotyped against the internal size standard of GeneScan-500 ROX. Then the genotypes of the 279 individuals were analysed in GeneMapper 4.1 (Applied Biosystems).

### Genotyping of SNPs in *OsZFAND3* gene, linkage and QTL mapping

To map the *OsZFAND3* gene on the previous linkage map^13^, all the 279 F_1_ individuals were used to genotype SNPs of *OsZFAND3*. Briefly, a targeted region of *OsZFAND3* gDNA was amplified using two primer sets: x3 SNP1 and x3 SNP2 (Supplementary Table S4). PCR amplification and programme for each individual sample were similar to those for the genotyping, except that the annealing temperature was 50 °C. The genotyping of identified SNPs was carried out by directly sequencing the PCR products in both directions with ABI 3730XL DNA sequencer (Applied Biosystems). SNPs among the resultant sequences were detected by alignment. Microsatellites Onil_1-89, Onil_19-15 and Onil_27-3, together with five microsatellites (Om251, Om432, Om188, Om287 and Om293) from Liu *et al*.^13^ were used for linkage mapping using the previous methods^41^,^42^,^43^. In order to determine whether *OsZFAND3* was localized on the reported sex-determing locus^13^, one of the identified SNPs was used to map *OsZFAND3* onto the linkage map using the method as described by Xia *et al*.^41^, and a LOD threshold of 3.0 was used to generate the Linkage map. Quantitative trait loci (QTL) analysis was carried out under BP model and LOD scores were obtained by bootstrapping with 10,000 permutations. QTL with LOD threshold of more than 95% (*P* < 0.05) at chromosome level were considered as significant.

### Analysis of tissue distribution of *OsZFAND3* with quantitative real-time RT-PCR (qPCR)

To analyze the tissue distribution of *OsZFAND3* mRNA, qPCR was used to determine the expression pattern. Total RNA from three males and three females was isolated using Trizol (Invitrogen, CA, USA), and treated with RNase-free DNase (Promega, WI, USA) for removal of contaminated gDNA. The A_260/280_ and A_260/230_ ratios of all the RNAs prepared were measured by Nanodrop 1000 spectrophotometer (Nanodrop Technologies, DE, USA), with the values within 1.80–2.00 and 2.00–2.50, respectively. The RNA quality was further accessed by electrophoresis on 2.0% agarose gel. The first strand of cDNA was synthesized using 5 μg of DNase-treated RNA by Reverse Transcriptase M-MLV Kit (Promega). The reactions in triplicates were performed using the KAPA^TM^ SYBR FAST qPCR Kit (KapaBiosystems, MA, USA) in an iQ^TM^ 5 Real Time PCR Detection System (Bio-Rad, CA, USA) according to the manufacturer’s instructions. The primers used for *OsZFAND3* amplification are shown in Supplementary Table S4. Relative gene expression was analyzed using the 2^−∆∆CT^ method^44^, and the housekeeping gene *β-actin* was selected to normalize the relative expression level of *OsZFAND3* in different tissues. One-way analysis of variance (ANOVA) with Tukey’s HSD test was performed using the SPSS statistics software package (SPSS, IL, USA)^45^,^46^. All results were expressed as mean + SE. Significance was accepted at the level of *P* < 0.05.

### Preparation of sense and anti-sense *OsZFAND3* cRNA probes and *in situ* hybridization

A 136-bp fragment of *OsZFAND3* transcript was amplified and sub-cloned into pGEM^®^-T Easy vector (Promega). The recombinant plasmid was linearized either with *Sac* II or *Spe* I (Promega). Anti-sense and sense RNA probes labelled were synthesized with a DIG RNA Labelling Kit (Roche Diagnostics, Germany) using the SP6 and T7 RNA polymerases (Roche), respectively. These probes were then treated with RNase-free DNase I (Promega) to remove the template DNA. *In situ* hybridization and histology was carried out as described previously^44^. Ovary and testis tissue sections (~5 μm) were hybridized with DIG-labeled antisense or sense RNA probes. Slides were mounted using Canada balsam (Sigma-Aldrich, MO, USA). The DIG was visualized using colorimetric substrate NBT/BCIP solutions (Roche) according to the manufacturer’s instructions and microscope images were captured through a light microscope (Lecia, Germany).

### Plasmid construction and subcellular localization

A pair of primers, containing restriction enzyme cutting sites of *Xho* I and *Hind* III, were used to amplify the ORF of *OsZFAND3*. The PCR products were cloned into expression vector pEGFP-N1 (Clontech Laboratories, CA, USA) and the recombinant plasmid (namely pEGFP-*OsZFAND3*) was confirmed by sequencing. To examine the subcellular localization of *OsZFAND3*, 2.2 × 10^5^ tilapia nerve fiber cells were transfected with 1 μg of plasmid pEGFP-*OsZFAND3* or the control vector pEGFP-N1, using Turbofect transfection reagent (Thermo) according to the manufacturer’s protocol. After 24 h transfection, cells were washed with PBS (pH 7.4), fixed with chilled methanol, stained with 6-diamidino-2-pheny-lindole (DAPI) and observed through a LSM 510 META inverted confocal microscope (Zeiss, Germany).

## Additional Information

**How to cite this article**: Ma, K. *et al*. Charactering the *ZFAND3* gene mapped in the sex-determining locus in hybrid tilapia (*Oreochromis spp*.). *Sci. Rep*. **6**, 25471; doi: 10.1038/srep25471 (2016).

## Supplementary Material

Supplementary Information

Supplementary Table S1

Supplementary Table S3

## References

[b1] Gamsjaeger R., Liew C. K., Loughlin F. E., Crossley M. & Mackay J. P. Sticky fingers: zinc-fingers as protein-recognition motifs. Trends Biochem. Sci. 32, 63–70 (2007). 17210253 10.1016/j.tibs.2006.12.007

[b2] Miller J., McLachlan A. D. & Klug A. Repetitive zinc-binding domains in the protein transcription factor IIIA from Xenopus oocytes. EMBO J. 4, 1609–1614 (1985). 4040853 10.1002/j.1460-2075.1985.tb03825.x PMC554390

[b3] Mackay J. P. & Crossley M. Zinc fingers are sticking together. Trends Biochem. Sci. 23, 1–4 (1998). 9478126 10.1016/s0968-0004(97)01168-7

[b4] Zarkower D. & Hodgkin J. Molecular analysis of the *C. elegans* sex-determining gene tra-1: A gene encoding two zinc finger proteins. Cell 70, 237–249 (1992). 1339311 10.1016/0092-8674(92)90099-x

[b5] Achermann J. C., Ito M., Hindmarsh P. C. & Jameson J. L. A mutation in the gene encoding steroidogenic factor-1 causes XY sex reversal and adrenal failure in humans. Nat. Genet. 22, 125–126 (1999). 10369247 10.1038/9629

[b6] Dalton J. E. . Male-specific Fruitless isoforms have different regulatory roles conferred by distinct zinc finger DNA binding domains. BMC Genomics 14, 659 (2013). 24074028 10.1186/1471-2164-14-659 PMC3852243

[b7] Huang J. . Expression analysis of rice A20/AN1-type zinc finger genes and characterization of ZFP177 that contributes to temperature stress tolerance. Gene 420, 135–144 (2008). 18588956 10.1016/j.gene.2008.05.019

[b8] Dixit V. M. . Tumor necrosis factor-alpha induction of novel gene products in human endothelial cells including a macrophage-specific chemotaxin. J. Biol. Chem. 265, 2973–2978 (1990). 2406243

[b9] Rebagliati M. R., Weeks D. L., Harvey R. P. & Melton D. A. Identification and cloning of localized maternal RNAs from Xenopus eggs. Cell 42, 769–777 (1985). 2414011 10.1016/0092-8674(85)90273-9

[b10] de Luis O., López-Fernández L. A. & del Mazo J. Tex27, a gene containing a zinc-finger domain, is up-regulated during the haploid stages of spermatogenesis. Exp. Cell Res. 249, 320–326 (1999). 10366431 10.1006/excr.1999.4482

[b11] Keaton J. M. . A comparison of type 2 diabetes risk allele load between African Americans and European Americans. Hum. Genet. 133, 1487–1495 (2014). 25273842 10.1007/s00439-014-1486-5 PMC4225163

[b12] Otake S., Endo D. & Park M. K. Molecular characterization of two isoforms of ZFAND3 cDNA from the Japanese quail and the leopard gecko, and different expression patterns between testis and ovary. Gene 488, 23–34 (2011). 21914466 10.1016/j.gene.2011.08.021

[b13] Liu F. . A microsatellite-based linkage map of salt tolerant tilapia (*Oreochromis mossambicus* x *Oreochromis spp*.) and mapping of sex-determining loci. BMC Genomics 14, 58 (2013). 23356773 10.1186/1471-2164-14-58 PMC3565888

[b14] Lim C. E. & Webster C. D. Tilapia: biology, culture, and nutrition. (Food Products Press, 2006).

[b15] Beardmore J., Mair G. & Lewis R. Monosex male production in finfish as exemplified by tilapia: applications, problems, and prospects. Aquaculture 197, 283–301 (2001).

[b16] Lee B. Y., Hulata G. & Kocher T. D. Two unlinked loci controlling the sex of blue tilapia (*Oreochromis aureus*). Heredity 92, 543–549 (2004). 15100706 10.1038/sj.hdy.6800453

[b17] Cnaani A. . Genetics of sex determination in tilapiine species. Sex. Dev. 2, 43–54 (2008). 18418034 10.1159/000117718

[b18] Eshel O., Shirak A., Weller J. I., Hulata G. & Ron M. Linkage and physical mapping of sex region on LG23 of Nile tilapia (*Oreochromis niloticus*). G3: Genes Genomes Genet. 2, 35–42 (2012). 10.1534/g3.111.001545 PMC3276181 22384380

[b19] Lee B. Y. . A second-generation genetic linkage map of tilapia (*Oreochromis spp*.). Genetics 170, 237–244 (2005). 15716505 10.1534/genetics.104.035022 PMC1449707

[b20] Palaiokostas C. . Mapping and validation of the major sex-determining region in Nile tilapia (*Oreochromis niloticus* L.) using RAD sequencing. Plos One 8, e68389 (2013). 23874606 10.1371/journal.pone.0068389 PMC3708939

[b21] Gammerdinger W. J., Conte M. A., Acquah E. A., Roberts R. B. & Kocher T. D. Structure and decay of a proto-Y region in Tilapia, *Oreochromis niloticus*. BMC Genomics 15, 975 (2014). 25404257 10.1186/1471-2164-15-975 PMC4251933

[b22] Li M. H. . A tandem duplicate of anti-müllerian hormone with a missense SNP on the Y chromosome is essential for male sex determination in Nile tilapia, *Oreochromis niloticus*. Plos Genet. 11, e1005678 (2015). 26588702 10.1371/journal.pgen.1005678 PMC4654491

[b23] Briknarová K. . The serine-rich domain from Crk-associated substrate (p130cas) is a four-helix bundle. J. Biol. Chem. 280, 21908–21914 (2005). 15795225 10.1074/jbc.M501258200

[b24] Vij S. & Tyagi A. K. A20/AN1 zinc-finger domain-containing proteins in plants and animals represent common elements in stress response. Funct. Integr. Genomics 8, 301–307 (2008). 18320246 10.1007/s10142-008-0078-7

[b25] Emerson R. O. & Thomas J. H. Adaptive evolution in zinc finger transcription factors. Plos Genet. 5, e1000325 (2009). 19119423 10.1371/journal.pgen.1000325 PMC2604467

[b26] Kikuchi K. & Hamaguchi S. Novel sex-determining genes in fish and sex chromosome evolution. Dev. Dyn. 242, 339–353 (2013). 23335327 10.1002/dvdy.23927

[b27] Hattori R. S. . A Y-linked anti-Müllerian hormone duplication takes over a critical role in sex determination. Proc. Natl. Acad. Sci. USA 109, 2955–2959 (2012). 22323585 10.1073/pnas.1018392109 PMC3286941

[b28] Smith C. A. . The avian Z-linked gene DMRT1 is required for male sex determination in the chicken. Nature 461, 267–271 (2009). 19710650 10.1038/nature08298

[b29] Myosho T. . Tracing the emergence of a novel sex-determining gene in medaka, Oryzias luzonensis. Genetics 191, 163–170 (2012). 22367037 10.1534/genetics.111.137497 PMC3338257

[b30] Koopman P., Gubbay J., Vivian N., Goodfellow P. & Lovell-Badge R. Male development of chromosomally female mice transgenic for Sry. Nature 351, 117–121 (1991). 2030730 10.1038/351117a0

[b31] Carrasco L. A. P., Penman D. J. & Bromage N. Evidence for the presence of sex chromosomes in the Nile tilapia (*Oreochromis niloticus*) from synaptonemal complex analysis of XX, XY and YY genotypes. Aquaculture 173, 207–218 (1999).

[b32] Campos-Ramos R. . Identification of putative sex chromosomes in the blue tilapia, *Oreochromis aureus*, through synaptonemal complex and FISH analysis. Genetica 111, 143–153 (2001). 11841163 10.1023/a:1013707818534

[b33] Yano A. . An immune-related gene evolved into the master sex-determining gene in rainbow trout, Oncorhynchus mykiss. Curr. Biol. 22, 1423–1428 (2012). 22727696 10.1016/j.cub.2012.05.045

[b34] Ganesh K. . CTNNBL1 is a novel nuclear localization sequence-binding protein that recognizes RNA-splicing factors CDC5L and Prp31. J. Biol. Chem. 286, 17091–17102 (2011). 21385873 10.1074/jbc.M110.208769 PMC3089553

[b35] Desmond C. R., Atwal R. S., Xia J. & Truant R. Identification of a karyopherin β1/β2 proline-tyrosine nuclear localization signal in huntingtin protein. J. Biol. Chem. 287, 39626–39633 (2012). 23012356 10.1074/jbc.M112.412379 PMC3501053

[b36] She Z. Y. & Yang W. X. SOX family transcription factors involved in diverse cellular events during development. Eur. J. Cell Biol. 94, 547–563 (2015). 26340821 10.1016/j.ejcb.2015.08.002

[b37] Larkin M. A. . Clustal W and Clustal X version 2.0. Bioinformatics 23, 2947–2948 (2007). 17846036 10.1093/bioinformatics/btm404

[b38] Tamura K., Stecher G., Peterson D., Filipski A. & Kumar S. MEGA6: molecular evolutionary genetics analysis version 6.0. Mol. Biol. Evol. 30, 2725–2729 (2013). 24132122 10.1093/molbev/mst197 PMC3840312

[b39] Xu B. & Yang Z. H. PAMLX: A graphical user interface for PAML. Mol. Biol. Evol. 30, 2723–2724 (2013). 24105918 10.1093/molbev/mst179

[b40] Yue G. H. & Orban L. A simple and affordable method for high-throughput DNA extraction from animal tissues for polymerase chain reaction. Electrophoresis 26, 3081–3083 (2005). 16047311 10.1002/elps.200410411

[b41] Xia J. H. . Whole genome scanning and association mapping identified a significant association between growth and a SNP in the IFABP-a gene of the Asian seabass. BMC Genomics 14, 295 (2013). 23634810 10.1186/1471-2164-14-295 PMC3653795

[b42] Xia J. H. . Mapping quantitative trait loci for omega-3 fatty acids in Asian seabass. Mar. Biotechnol. 16, 1–9 (2014). 23887675 10.1007/s10126-013-9524-1

[b43] Liu F. . A genome scan revealed significant associations of growth traits with a major QTL and GHR2 in tilapia. Sci. Rep. 4, 7256 (2014). 25435025 10.1038/srep07256 PMC4248272

[b44] Ma K. Y., Liu Z. Q., Lin J. Y., Li J. L. & Qiu G. F. Molecular characterization of a novel ovary-specific gene fem-1 homolog from the oriental river prawn, Macrobrachium nipponense. Gene 575, 244–252 (2016). 26367327 10.1016/j.gene.2015.08.070

[b45] Hsieh Y. C., Lin T. L., Lin C. M. & Wang J. T. Identification of PblB mediating galactose-specific adhesion in a successful *Streptococcus pneumoniae* clone. Sci. Rep. 5, 12265 (2015). 26193794 10.1038/srep12265 PMC4508584

[b46] Hu Q., Guo W., Gao Y., Tang R. & Li D. P. Molecular cloning and analysis of gonadal expression of Foxl2 in the rice-field eel *Monopterus albus*. Sci. Rep. 4, 6884 (2014). 25363394 10.1038/srep06884 PMC4217102

