# Supplementary material for: Charactering the ZFAND3 gene mapped in the sex-determining locus in hybrid tilapia (Oreochromis spp.)
Source: Sci Rep. 2016 May 3;6:25471. doi: 10.1038/srep25471 (PMC4853787; doi:10.1038/srep25471)
Supplement: Supplementary Information [file srep25471-s1.pdf]

Supplementary files for the paper

# Charactering the *ZFAND3* gene and mapping it in the sex-determining locus in hybrid tilapia (*Oreochromis spp*.)

**Keyi Ma[1], Minghui Liao[1], Feng Liu[1], Baoqing Ye[1], Fei Sun[1], & Gen Hua Yue [1,2,3*]**

[1]Molecular Population Genetics and Breeding Group, Temasek Life Sciences Laboratory, 1 Research Link, National University of Singapore, Singapore 117604, Republic of Singapore. [2]Department of Biological Sciences, National University of Singapore, 14 Science Drive 4, Singapore 117543, Republic of Singapore. [3]School of Biological Sciences, Nanyang Technological University, 60 Nanyang Drive, Singapore 637551, Republic of Singapore

[*]Corresponding author

Dr. G.H. Yue

Temasek Life Sciences Laboratory, 1 Research Link, National University of Singapore, 117604 Singapore, Republic of Singapore.

Tel.: + 65 68727405. Fax: + 65 68727007. Email: genhua@tll.org.sg

A

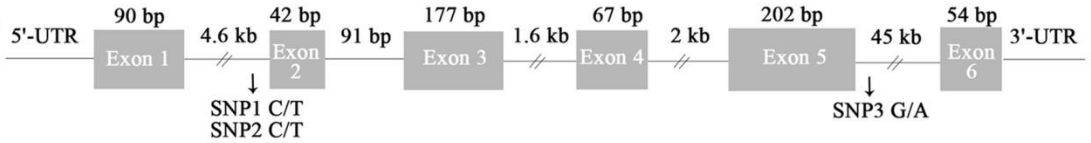


B

```
  1 acacacttcccccctctctgctagtggttcgttcctcactccagctgatcggcccaggatccccggtggtttcgtccgttgctggctttga
 91 cggccccagcgtcttaacgtcgacttcaactactttaatttgacggacttggtggcggcttgtgtcgtcaaattatctaccctacactgc
181 aactcggaagaattatctgggcagagtgacggttgtatgcttatcaaagccaaggggatctaaacgcaaatacgaggccctcactccatc
271 cacggattcacctgctacaccgacagcttgacacgggagacagacATGGGCGACACGGGGAGCGAGGGCAGCAAGCCTCCGAGTAACGTT
                                                 M  G  D  T  G  S  E  G  S  K  P  P  S  N  V   15
361 AACGTTATACCCCCTCGCTGCCCCTGTGGGTTCTGGGGGTCAAGCAAAACCATGAATCTCTGCTCAAAATGTTTTGCAGACACTCAAAAG
     N  V  I [P  P  R  C  P  C  G  F  W  G  S  S  K  T  M  N  L  C  S  K  C  F  A] D  T  Q  K   45
451 AAACAGCCAAACGACAACTGTGCTCCAAAGGCCTCTTCAAACTCTAACACCAGTCAAGCAGACATGTTCTGTAATGAAACAAACAACAGC
     K  Q  P  N  D  N  C  A  P  K  A  S  S  N  S  N  T  S  Q  A  D  M  F  C  N  E  T  N  N  S   75
541 ATTAGTCAGTCCCTCATATCAGAAGACCCTTTGCCCGGAGAGATAGGAGTGACATCTGTACCTGTTCAGGACGGAACATCCAGCAACGAC
     I  S  Q  S  L  I  S  E  D  P  L  P  G  E  I  G  V  T  S  V  P  V  Q  D  G  T  S  S  N  D  105
631 ACAGTCTTGGGTTCATTCTCCACTCCGACAAAACGTACATTTGAGTCAGCCTCAGATTCAGAAAGTGACGTTTCACCTGAAAAGCGAGCG
     T  V  L  G  S  F  S  T  P  T  K  R  T  F  E  S  A  S  D  S  E  S  D  V  S  P  E  K  R  A  135
721 AGAGTAGGTGGGATCCCAGAGGGTGAGGAGTCTTCATCATCATCATCGTCTTCATCCTCGTCTCGCAGTGGCTCTAAACAGCGGAGCCGT
     R  V  G  G  I  P  E  G  E  E  S  S  S  S  S  S  S  S  S  S  S  R  S  G  S  K  Q  R  S  R  165
811 AAGCGTTGCCATCGCTGCCAAACCAAACTGGAGCTGGTACAGCAAGAGCTGGGTTCCTGTCGCTGTGGTTGTTCCTACAACATGTTGTCT
     K  R  C  H  R  C  Q  T  K  L  E  L  V  Q  Q  E  L  G  S  C  R  C  G  C  S  Y  N  M  L  S  195
901 TTTGAGATTCATGGCCTTAAGCAGCAATGAtgcgctctcagcaagacatgccaagactgctggctccaacccaacatgaggggcaaaaac
     F  E  I  H  G  L  K  Q  Q  *                                                               204
991 gaaaggaaggaaggaaagagacacatacttgagccagaacccatgattttgtttactctg
```

**Supplementary Figure S1. Genomic structure (A) and deduced amino acid sequence (B) of the *OsZFAND3* gene**

A: horizontal lines and boxes indicate introns and exons in genomic structure, respectively. The locations of the three SNPs are marked with arrows. B: nucleotides (top) are numbered from 5' to 3', starting from the initiator codon (ATG), while amino acid residues (bottom) are numbered starting with the first methionine (M) residue. The asterisk (*) indicates the stop codon (TGA). A20-like zinc finger region and Serine-rich region are boxed and underline, respectively.

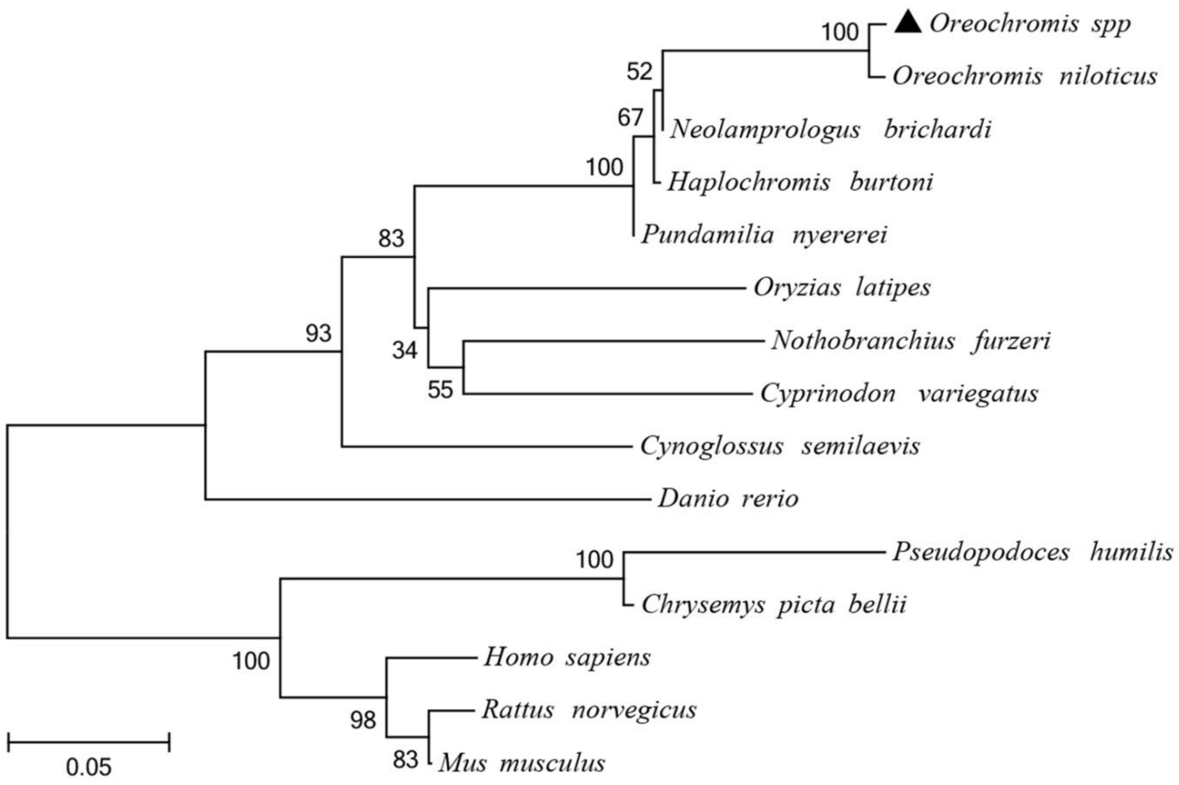


**Supplementary Figure S2. An unrooted phylogenetic tree of the *ZFAND3* gene**

The phylogenetic tree was inferred by using the Neighbor-Joining method with whole deduced amino acid sequences. The values at the forks are Neighbor-Joining bootstrap supporting data. For the branch length, the distance given in the scale represents 0.05 amino acid substitution/site.

**Supplementary Table S1**

**Summary of genotypes of parents and their $F_1$ progenies of tilapia at SNP1, SNP2, SNP3, Onil_1-89, Onil_19-15 and Onil_27-3 reported in this study** (Please online **Supplementary Dataset).**

**Supplementary Table S2**

**Amino acid identities (%) of the *ZFAND3* gene between hybrid tilapia and other representative species**

| **Gene** | **GenBank Accession No.** | **Amino acid** | **Identity** | ***P*-value** |
|---|---|---|---|---|
| *Oreochromis niloticus* | XP_005447615.1 | 204 | 99% | 2e-149 |
| *Neolamprologus brichardi* | XP_006779955.1 | 238 | 93% | 5e-123 |
| *Haplochromis burtoni* | XP_005934639.1 | 238 | 93% | 2e-122 |
| *Cynoglossus semilaevis* | XP_008309070.1 | 254 | 69% | 5e-96 |
| *Homo sapiens* | NP_068762.1 | 227 | 52% | 2e-58 |
| *Rattus norvegicus* | NP_001012175.1 | 227 | 51% | 2e-57 |
| *Mus musculus* | NP_683728.1 | 205 | 47% | 4e-50 |
| *Pseudopodoces humilis* | XP_005533586.1 | 223 | 50% | 1e-51 |
| *Chrysemys picta bellii* | XP_008172961.1 | 210 | 45% | 5e-47 |
| *Pundamilia nyererei* | XP_005733397.1 | 216 | 82% | 1e-93 |
| *Oryzias latipes* | XP_004067319.1 | 244 | 72% | 4e-86 |
| *Nothobranchius furzeri* | XP_015808368.1 | 247 | 70% | 3e-76 |
| *Cyprinodon variegatus* | XP_015233722.1 | 248 | 72% | 7e-73 |
| *Danio rerio* | NP_001025346.1 | 226 | 58% | 1e-54 |

**Supplementary Table S3**

**Evolution estimates of *ZFAND3* gene by dN/dS values using maximum likelihood analysis** (Please online **Supplementary Dataset).**

**Supplementary Table S4**

**Summary of primer sets used for mapping and characterizing the *OsZFAND3* gene.**

| **Marker** | **Primer sequence (**5'-3'**)** | **Ta (**°C**)** | **Purpose** |
|---|---|---|---|
| Onil_1-89 | TCTCGCGGATTCTCTGCTC | 55 | mapping |
| | TTGCCATTGTCTTCCACTGTAA | | |
| Onil_19-15 | CACCTACAATTCTAGCACATACCA | 55 | mapping |
| | GAATTTCTTCAAGGACACTGTCA | | |
| Onil_27-3 | CCTGCAGTGGGTTTGGATT | 55 | mapping |
| | AGTCAGCGATAGAAGAGGGAATAC | | |
| x3 SNP1 | CCCCCAGACGCACTTATG | 50 | SNP |
| | AGAGGCCTTTGGAGCACAG | | |
| x3 SNP2 | CATCGCTGCCAAACCAAACT | 50 | SNP |
| | TTCCGCCCCCAATACAATCT | | |
| β-actin | TGACCCAGATCATGTTCGAGAC | 60 | qPCR |
| | GTGGTGGTGAAGGAGTAGCC | | |
| x3 | GCTGGGTTCCTGTCGCTGTG | 60 | qPCR, ISH |
| | TCATGGGTTCTGGCTCAAGTATG | | |
| x3-pc | AG*CTCGAG*ATGGGCGACACGGGGAGC | 60 | Localization |
| | CC*AAGCTT*TTGCTGCTTAAGGCAATGAATCTCA | | |

Note: the letters in italics were restriction sites. The letters underlined were protective bases.
